# Reduction of leptin levels by four cardiac hormones: Implications for hypertension in obesity

**DOI:** 10.3892/etm.2013.1173

**Published:** 2013-06-21

**Authors:** MEGHAN L. LANE, DAVID L. VESELY

**Affiliations:** Departments of Medicine, Molecular Pharmacology and Physiology, and James A. Haley VA Medical Center, University of South Florida Morsani School of Medicine, Tampa, FL 33612, USA

**Keywords:** leptin, hypothalamus, hypertension in obesity, cardiac hormones

## Abstract

Circulating levels of leptin are increased in obesity and have been proposed to contribute to the development of hypertension in obese individuals. Four cardiac hormones, specifically, vessel dilator, long-acting natriuretic peptide (LANP), kaliuretic peptide and atrial natriuretic peptide (ANP), have blood pressure-lowering properties and correlate with the presence of hypertension in obesity. The objective of this study was to determine whether one or more of these cardiac hormones was able to decrease the levels of leptin in the hypothalamus, an area of the brain that has been demonstrated to synthesize more than 40% of leptin in the circulation. The effects of these four cardiac hormones on leptin were examined using dose-response curves in the rat hypothalamus, which synthesizes leptin. Vessel dilator, LANP, kaliuretic peptide and ANP maximally decreased the levels of leptin in hypothalamic cells by 79, 76, 80 and 62%, respectively (P<0.0001 for each). The cardiac hormones decreased leptin levels over a concentration range of 100 pM to 10 *μ*M, with the most significant reductions in leptin levels occurring when the concentrations of the hormones were at micromolar levels. The results of the study suggest that the four cardiac hormones lead to significant reductions in hypothalamic leptin levels, which may be an important mechanism for alleviating leptin-induced hypertension in obesity.

## Introduction

Leptin is a 16 kDa-peptide (167 amino acids) that is synthesized and secreted predominantly by white adipose tissue (1). One of the major effects of this hormone is the control of energy balance via the hypothalamus, which was previously considered to be mediated through binding to leptin receptors in the hypothalamus (2–4). However, leptin levels are higher in the blood leaving the brain than in that entering it, suggesting that leptin may be synthesized in the hypothalamus and other brain tissues (5). More than 40% of circulating leptin originates from the brain in healthy males (6). Furthermore, the plasma leptin concentrations contributed by the brain demonstrate a six-fold increase in obese males compared with healthy males (935±32 ng/ml in obese versus 160±59 ng/ml in healthy males) (6). Leptin is synthesized in the hypothalamus (7). Leptin mRNA expression has been detected in the hypothalamus and this expression of leptin has been demonstrated to be suppressed by 48 h of fasting (7). In the hypothalamus, the leptin gene is, thus, regulated by nutrient availability. The detection of leptin expression in the hypothalamus by reverse transcription-polymerase chain reaction (RT-PCR) has been confirmed with sequence analysis using rat hypothalami (7). Leptin protein has also been demonstrated to be present in rat hypothalami by immunocytochemistry (7). Therefore, leptin gene expression and leptin itself are present in the hypothalamus.

Although leptin has been suggested to reduce appetite, obese individuals generally exhibit high circulating leptin levels (8) and, as mentioned previously, a large proportion of the high leptin levels that are apparent in obesity originate in the brain, with six-fold more leptin secreted from the brain into the circulation of obese versus healthy individuals (6). It has been suggested that these high circulating levels of leptin in obesity function pathophysiologically for the development of hypertension (8). Epidemiological studies have indicated that 65–75% of the risk for hypertension is excess weight (9,10). Chronic increases in leptin levels result in a persistent elevation in mean arterial pressure and this hypertensive blood pressure is rapidly reversed with cessation of leptin administration (11). Acute infusions of leptin have also been demonstrated to lead to an abrupt increase in blood pressure (12). Similar increases in systolic blood pressure have been observed in transgenic mice overexpressing leptin (13).

The heart and adipose tissue are endocrine organs and studies have increasingly suggested that cross-talk exists between them, although the precise mechanism is poorly defined (14–19). The heart synthesizes four hormones, the products of a single gene, which have significant blood pressure-lowering effects (20). These cardiac hormones, which are vessel dilator, long-acting natriuretic peptide (LANP), atrial natriuretic peptide (ANP) and kaliuretic peptide, are vasodilators, with blood pressure-lowering properties in animals (21–26) and humans (27–29). The original hypothesis for hypertension was the presence of a defect in the production of the blood pressure-lowering ANPs (30,31). However, experimental data have revealed that, rather than being decreased, the levels of these blood pressure-lowering peptides are elevated in the circulation in an apparent attempt to overcome the elevated blood pressure (30–34). ANP levels are increased in essential hypertension (30,34). The hypertension in obesity is also associated with increased circulating concentrations of ANP (32), LANP (33) and vessel dilator (33), which decrease into the normal range when the high blood pressure is reduced by weight loss (32–34). In the present study, we hypothesized that since the levels of cardiac hormones correlate with blood pressure in obesity (32–34), the blood pressure-reducing effects of these hormones (20–29) may be mediated, in part, by decreased leptin production in the hypothalamus.

## Materials and methods

### Cardiac hormones

The cardiac hormones (vessel dilator, ANP, kaliuretic peptide and LANP) were obtained from Phoenix Pharmaceuticals, Inc., Belmont, CA, USA.

### Hypothalamic cells

Hypothalamic cells (ATCC no. CRL-2005; DI TNC1) were obtained from the American Type Culture Collection (ATCC) Manassas, VA, USA. The ATCC authenticated this cell line.

### Culture of hypothalamic cells

Propagation of the hypothalamic cells was performed in Dulbecco’s Modified Eagle’s medium with an addition of 10% heat-inactivated fetal bovine serum (Sigma Chemical Corporation, St. Louis, MO, USA) and 1% penicillin, streptomycin and fungizone at a temperature of 37°C, as recommended by the ATCC. The number of cells in culture was 1.44×10^6^ cells/ml. Cells were dispensed into new flasks with subculturing every 6 days. The growth medium was changed every 3 days.

### Leptin enzyme-linked immunosorbent assay (ELISA)

The Quantikine^®^ leptin immunoassay ELISA used to measure leptin levels was obtained from R&D Systems (Minneapolis, MN, USA). This 3.5 h solid phase ELISA contained *E. coli*-expressed recombinant leptin and antibodies raised against the recombinant leptin. This quantitative sandwich enzyme immunoassay utilized a monoclonal antibody specific for leptin. The immunoassay has been shown to quantitate recombinant leptin accurately. Results obtained by measuring natural leptin revealed that the dose-response curves obtained with the recombinant Quantikine^®^ assay paralleled the curves with natural leptin. The assay had a 98% recovery of leptin in the previously mentioned cell culture media. The minimal detectable concentration of leptin in this assay was 7.8 pg/ml. The levels of leptin measured are the amount in the cells plus the amount of leptin secreted into the media.

### Leptin protocol

The hypothalamic cells (1.44×10^6^ cells/ml) were subcultured for 24 h, prior to 50 *μ*l cell culture supernatant being added to 96-well plates with 50 *μ*l media, containing 100 pM, 1 nM, 10 nM, 100 nM, 1 *μ*M and 10 *μ*M of each of the four cardiac hormones, separately (n=9 for each concentration). The hypothalamic cells were subsequently evaluated using the leptin ELISA from R&D Systems. Following this, the mean of the nine measurements at each concentration of the respective peptide hormones was then calculated and this was compared with the mean of the leptin concentrations in the control hypothalami, which had not been exposed to any of the cardiac hormones. The leptin data are shown in the figures as the decrease in the leptin level (i.e., the percentage decrease) compared with the level of leptin in the untreated hypothalami. The standards from R&D Systems were added to the blank wells to serve as reference points for known leptin concentrations. In this assay, absorbance was recorded at a 540 nm wavelength using a 96-well BioTek Gen 5, Synergy Mx microplate reader (BioTek Instruments, Inc., Winooski, VA, USA). There were 48 hypothalamic controls in these experiments.

### Statistical analysis

Data are expressed as the mean ± standard error of the mean (SEM). Statistical analysis of the data were performed by one way analysis of variance (ANOVA) with a repeated measures design for within-group comparisons, using a statistical module of Excel software (Microsoft Corporation, Redmond, WA, USA). A value of P<0.05 was considered to indicate a statistically significant difference.

## Results

### Vessel dilator decreases the hypothalamic concentrations of leptin by up to 79%

**Figure 1.**
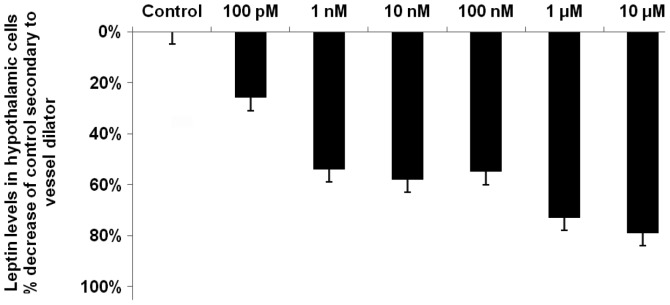
Vessel dilator decreases the hypothalamic concentration of leptin by up to 79%. Vessel dilator maximally decreased leptin levels in the hypothalamic cells by 79% (P<0.0001) at a concentration of 10 *μ*M in comparison with the control (85±4 pg/ml). Vessel dilator caused a significant reduction in leptin levels in the hypothalamus at each of its concentrations, with reductions of 26, 54, 58, 55 and 73% at concentrations of 100 pM, 1 nM, 10 nM, 100 nM and 1 *μ*M, respectively. These results were significant at P<0.001, with the exception of the 100 pM concentration (P<0.05), as demonstrated by analysis of variance (ANOVA) with a repeated measures design for within-group comparisons. n=9 for each concentration of vessel dilator; n=48 for controls.

Vessel dilator decreased the concentration of leptin by a maximum of 79% (P<0.0001) from the control value of 85±4 pg/ml. The maximal reduction was obtained when the highest concentration of vessel dilator, i.e., 10 *μ*M was used (Fig. 1). At the lowest concentration of the vessel dilator (100 pM), there was a 26% reduction in the concentration of leptin (P<0.05). The dose-response curves indicated that vessel dilator also decreased leptin levels by 54, 58, 55 and 73% at concentrations of 1, 10 and 100 nM and 1 *μ*M, respectively (P<0.001 for each; Fig. 1).

### LANP decreases hypothalamic leptin by up to 76%

**Figure 2.**
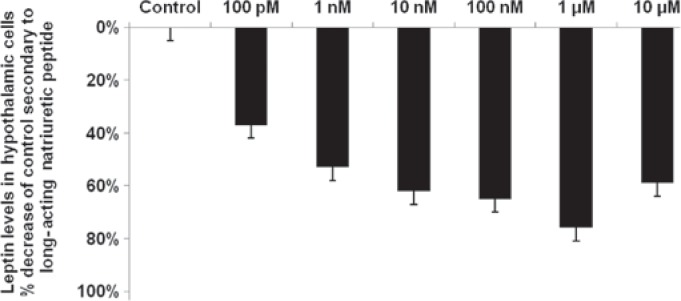
Long-acting natriuretic peptide (LANP) decreases hypothalamic leptin by up to 76%. LANP decreased leptin levels by 76% (P<0.0001) at a concentration of 1 *μ*M. LANP decreased leptin levels by 37, 53, 62, 65 and 59% at concentrations of 100 pM, 1 nM, 10 nM, 100 nM and 10 *μ*M, respectively. These results were significant at P<0.001, with the exception of the 100 pM concentration (P<0.01), as demonstrated by analysis of variance (ANOVA) with a repeated measures design for within-group comparisons. n=9 for each concentration of LANP; n=48 for controls.

LANP decreased leptin levels by up to 76% (P<0.0001) in the hypothalami, with the maximal reduction occurring at a LANP concentration of 1 *μ*M (Fig. 2). LANP, similar to vessel dilator, decreased leptin by the smallest amount at its lowest concentration, 100 pM; however, this 37% reduction in leptin was significant at P<0.01. There was a significant (P<0.001) reduction in leptin levels at each of the other concentrations of LANP, with reductions of 53, 62, 65 and 59% at concentrations of 1, 10 and 100 nM and 10 *μ*M LANP, respectively (Fig. 2).

### Reduction of leptin levels in the hypothalami secondary to ANP

**Figure 3.**
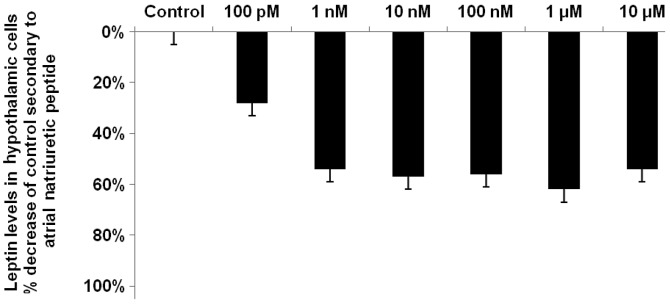
Atrial natriuretic peptide (ANP) decreases the hypothalamic leptin level by up to 62%. ANP decreased leptin levels by 62% (P<0.0001) at a concentration of 1 *μ*M. ANP decreased leptin levels by 28, 54, 57, 56 and 54% at concentrations of 100 pM, 1 nM, 10 nM, 100 nM and 10 *μ*M, respectively. These reductions in leptin levels were significant at P<0.001, with the exception of the 100 pM concentration (P<0.05), as demonstrated by analysis of variance (ANOVA) with a repeated measures design for within-group comparisons. n=9 for each concentration of ANP; n=48 for controls.

ANP, like LANP, caused its maximal reduction (62%; P<0.0001) in hypothalamic leptin levels at a concentration of 1 *μ*M and its smallest reduction (28%; P<0.05) at a concentration of 100 pM (Fig. 3). There was a significant reduction in leptin levels at all concentrations of ANP, with reductions of 54, 57, 56 and 54% at concentrations of 1, 10 and 100 nM and 10 *μ*M, respectively (P<0.001 for each concentration).

### Kaliuretic peptide decreases hypothalamic leptin by up to 80%

**Figure 4.**
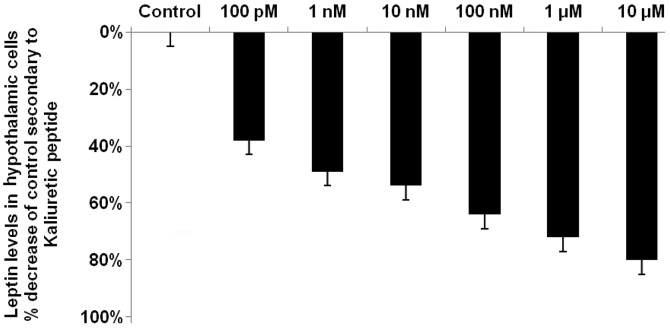
Kaliuretic peptide decreases hypothalamic leptin concentrations by up to 80%. Kaliuretic peptide decreased the leptin level by 80% (P<0.0001) at a concentration of 10 *μ*M. Kaliuretic peptide decreased leptin levels by 35, 49, 54, 64 and 72% at concentrations of 100 pM, 1 nM, 10 nM, 100 nM and 1 *μ*M, respectively. These results were significant at P<0.001, with the exception of the 100 pM concentration (P<0.01), as demonstrated by analysis of variance (ANOVA) with a repeated measures design for within-group comparisons. n=9 for each concentration of kaliuretic peptide; n=48 for controls.

Kaliuretic peptide decreased leptin levels by up to 80% (P<0.0001), with a maximal reduction occurring at the highest concentration of kaliuretic peptide, i.e., 10 *μ*M (Fig. 4). Kaliuretic peptide also caused a significant (P<0.0001) 72% reduction in leptin levels at a concentration of 1 *μ*M. Kaliuretic peptide significantly decreased leptin levels at each of its concentrations in the dose-response curves, with reductions of 35, 49, 54 and 64% at concentrations of 100 pM and 1, 10 and 100 nM (P<0.001 for each, with the exception of the 100 pM concentration where P<0.01). Thus, with respect to the maximal reduction in leptin levels, the effects of vessel dilator, LANP and kaliuretic peptide were approximately equal and each of these cardiac hormones had a stronger ability than ANP to decrease leptin levels.

## Discussion

Vessel dilator, LANP, kaliuretic peptide and ANP each significantly decreased leptin levels in the hypothalamus, an area of the brain that synthesizes leptin (7). The brain contributes more than 40% of the leptin in the circulation (6). The hypothalamus and brain contribute approximately six-fold more to the circulating concentration of leptin in obese individuals in comparison with the concentration in the circulation of healthy individuals (6), which may be the reason that leptin is elevated in the circulation of obese individuals (8). This suggests that there is an increase of leptin being synthesized in the hypothalamus in obese individuals. Furthermore, this indicates that the hypothalamus is significant in the elevation of leptin levels in the circulation of obese individuals with hypertension (6). With regard to hypertension in obesity, the present results indicated that the hypertension may be treated with the vasodilatory cardiac hormones investigated in the present study, since increased levels of leptin are correlated with the development of hypertension in obese individuals (8,11,12). The ability of all four cardiac hormones to markedly decrease leptin levels was suggestive of a novel potential treatment target for hypertension in obesity, since these four cardiac hormones have demonstrated blood pressure-lowering properties (21–29). The circulating concentrations of these four cardiac hormones increase in individuals with high blood pressure in an apparent attempt to overcome the constriction of the blood vessels (34). In calorie-restricted weight reduction, the four cardiac hormones have been demonstrated to be correlated in a linear fashion (P<0.0001) with blood pressure reduction (34). During this blood pressure reduction, plasma aldosterone and plasma renin were inversely correlated with the concentration of the four cardiac hormones and blood pressure (34). These results were consistent with the ability of ANP to inhibit renin release and aldosterone secretion from the adrenal gland, as well as the strong inhibition of renin release mediated by vessel dilator (25,34). The ability of these hormones to decrease leptin levels thus suggests one mechanism for the known correlation with blood pressure in obesity (32–34).

In the present study, the cardiac hormones were demonstrated to directly decrease leptin levels in the hypothalamus. It may be expected that the cardiac hormones also have the ability to decrease leptin levels in other leptin-synthesizing tissues, as leptin promotes angiogenesis by increasing vascular endothelial growth factor (VEGF) levels (35) and the cardiac hormones have been demonstrated to decrease levels of VEGF and the VEGFR-2 receptor by up to 92% (36). Thus, one of the mediators (VEGF) by which leptin causes vascular permeability and angiogenesis (35) is inhibited by each of the cardiac hormones (36). This suggests that the effects of leptin on blood vessels may also be decreased by the four cardiac hormones. In addition, ANP has been shown to inhibit leptin release from adipose tissues (37), with receptors for ANP being present in adipose tissues (38).

Hormone-sensitive lipase breaks down triglycerides into non-essential fatty acids and glycerol (14). This hydrolysis is commonly termed lipolysis (14). ANP activates hormone-sensitive lipase through an increase in cyclic guanosine 3′,5′-monophosphate (cGMP) production, via the enhancement of guanylyl cyclase (15). Furthermore, the three other cardiac hormones synthesized by the ANP prohormone gene also markedly enhance cGMP production by stimulating guanylyl cyclase (39). The application of ANP via a microdialysis probe has been shown to increase lipolysis in abdominal subcutaneous adipose tissue of healthy young males (14,16), while a systemic ANP infusion increases lipolysis (17,18), even at physiological concentrations (19). Prior to the demonstration that cardiac hormones were able to cause lipid mobilization, catecholamines and insulin were considered the major acute regulators of lipid mobilization and they act via a cyclic adenosine 5′-phosphate (AMP)-dependent regulation of lipolysis (14). By contrast, the cardiac hormones activate the guanylyl cyclase-cGMP pathway (15,20,39), which is completely independent from the cyclic AMP-dependent pathway in adipose cells (15). Resistance to catecholamine-induced lipolysis in subcutaneous adipose tissue has been demonstrated in obese adults (40) and obese children (41). Since the cardiac hormones increase lipolysis in obese subjects, as well as helping to alleviate obesity-interrelated hypertension mediated by leptin, this suggests they may be a multi-targeted novel therapy for obesity.
